# Evaluating the Impact of Assistive Technologies on Individuals With Disabilities in Benin: Protocol for a Cross-Sectional Study

**DOI:** 10.2196/60869

**Published:** 2024-12-09

**Authors:** Orthelo Léonel Gbètoho Atigossou, Sègbédji Joseph Martial Capo-chichi, Penielle Mahutchegnon Mitchaї, Aristide S Honado

**Affiliations:** 1 Center for Interdisciplinary Research in Rehabilitation and Social Integration Centre Intégré Universitaire de Santé et de Services Sociaux de la Capitale Nationale Quebec, QC Canada; 2 School of Rehabilitation Sciences Faculty of Medicine Université Laval Quebec, QC Canada; 3 Direction Générale de la Médecine Hospitalière et des Explorations Diagnostiques Ministère de la Santé Cotonou Benin; 4 Service de kinésithérapie Centre Hospitalier Départemental du Mono et du Couffo Lokossa Benin

**Keywords:** assistive technologies, assistive technology assessment, individuals with disabilities, disabilities, cross-sectional study, well-being, quality of life, effects, Benin

## Abstract

**Background:**

A significant proportion of individuals with disabilities in resource-limited countries require at least 1 assistive technology (AT) device to enhance their functioning and autonomy. However, there is limited evidence regarding the actual needs of AT users in these regions concerning the adequacy of ATs.

**Objective:**

This research aims to assess the effects of ATs on AT users in a resource-limited country.

**Methods:**

A cross-sectional study will be conducted in Benin, a sub-Saharan African country, using a nonprobability sample of AT users. Participants will undergo evaluation using standardized tools to assess their psycho-affective status, satisfaction with ATs, perception of the functional effects of ATs, well-being, and quality of life. Additionally, a survey based on the World Health Organization's rATA (rapid assistive technology assessment) tool will be conducted to gather sociodemographic and other data concerning the use of ATs. The findings will be organized and discussed using the Consortium on Assistive Technology Outcomes Research taxonomy, focusing on aspects related to the effectiveness and social significance of ATs, as well as the subjective well-being of AT users.

**Results:**

The process of identifying potential participants began in August 2024, and data collection is scheduled to start in January 2025 and continue for 12 months.

**Conclusions:**

This research will provide an overview of the effects induced by the use of ATs, as well as describe the profile of AT users in Benin. To our knowledge, this will be the first study to examine the impact of ATs in Benin. It will therefore make a significant contribution to the existing data on the use of ATs in sub-Saharan Africa.

**International Registered Report Identifier (IRRID):**

PRR1-10.2196/60869

## Introduction

The global population of individuals with disabilities is rapidly increasing, and assistive technologies (ATs) are emerging as substantial supports for these individuals [1,2]. ATs facilitate the social inclusion and involvement of people who are disabled [2], while also enabling them to maintain an optimal level of independence. Accordingly, the World Health Organization (WHO) has recognized access to appropriate ATs as a fundamental human right for all individuals who require them [2,3]. In 2022, a joint report by the WHO and the UNICEF (United Nations Children’s Fund) estimated that over 2.5 billion individuals living with various disabilities required at least 1 AT to improve their functioning and autonomy [2]. Additionally, this report states that most of these individuals reside in low- and middle-income countries, particularly in sub-Saharan Africa [2]. Unfortunately, only a small percentage—ranging from 5% to 15%—of these individuals have actual access to ATs [4]. Based on a recent analysis of 252 studies carried out in resource-limited countries, the provision and supply of ATs appear to be disproportionately influenced by the type or function of the AT [4]. For instance, there appears to be a lower availability of ATs designed for hearing and communication compared to those designed for mobility [4]. The lack of financial resources and unstructured institutional policies are the main reasons for the observed issues with the accessibility and availability of ATs in these countries [4,5].

National and international nongovernmental charities are working to provide ATs to disadvantaged populations [2,6]. However, a significant proportion of these ATs are often misused or abandoned by beneficiaries due to insufficient consideration of their actual needs [2,6]. Similarly, there is limited literature on the impact of ATs on the quality of life (QoL) and well-being of users, especially in resource-limited regions.

It follows that it is essential to conduct comprehensive in-depth documentation of the effects of ATs to address concerns regarding the paucity of data on ATs.

Most conceptual frameworks or taxonomies designed to understand the effects of using ATs describe these effects as multifaceted and multidimensional, considering both the users and their environments [7]. For instance, the Consortium on Assistive Technology Outcomes Research (CATOR) provided a taxonomy that covers 3 broad domains—effectiveness, social significance, and subjective well-being—in which the impacts of ATs can be classified [8]. The domains of this taxonomy cover various objective and subjective aspects of an AT user’s overall life.

Thus, ATs can help individuals with disabilities to perform daily tasks and personal care activities [2,3,9,10]. ATs can reduce the difficulties experienced by these individuals, improve their QoL, and broaden the range of useful and enjoyable activities in which they wish to participate [2]. For example, ATs such as wheelchairs and walking aids (canes or walkers) are designed to enhance mobility and promote accessibility for individuals with disabilities in various environments [11]. Moreover, ATs can reduce the workload of family caregivers of individuals who are disabled [12-14].

The advantages and disadvantages of ATs are generally assessed based on specific indicators that can predict the QoL and overall well-being of AT users [15,16]. These indicators include satisfaction [17], the psychosocial impact of AT use [18], and the perception of functional benefits [19]. Furthermore, they provide information on the opinions of AT users concerning their expectations regarding the use of AT. More specifically, they provide information on the impact of AT on users’ daily lives per functional independence, performance, productivity, willingness to try new things, ability to take risks or take advantage of life’s opportunities, self-confidence, and ability to adapt to life [19-21]. Numerous studies in high-income countries have used them to predict long-term AT use or AT abandonment [22-26]. In sub-Saharan Africa, very few studies have focused on some of the indicators described above [21,27,28].

To our knowledge, there is no data on the use of ATs in Benin, nor on such indicators, despite the increase in the number of people who are disabled recorded in successive censuses [29]. Thus, understanding the real needs of these individuals per the adequacy of ATs in this country remains a genuine challenge. This research aims to document the literature on this issue by evaluating these indicators among AT users in Benin.

## Methods

### Study Design, Participants, and Recruitment

A cross-sectional study will support this research project, using a nonprobability sample of AT users in Benin. The participants will be evaluated by multiple teams of recruiters, with two recruiters assigned to each department in Benin. Recruiters will be selected based on specific criteria: (1) being a health professional, (2) having experience in participant recruitment, and (3) fluency in French and one of the local languages spoken in the covered area. They will receive training on the questionnaires and assessment methods that will be used in this study. Participants will be identified through the Fédération des Associations des Personnes Handicapées du Bénin (FAPHB). FAPHB is a national network that brings together all associations of individuals who are disabled, whether they use ATs or not, residing in one of Benin’s regions or departments. FAPHB maintains a register of individuals who are disabled and who are regularly identified as such. A formal request will be made to FAPHB to establish contact with those among them who use ATs. In addition, participants will be recruited from hospital rehabilitation units and community rehabilitation centers across various regions in Benin. Before recruitment, authorization will be requested from these centers. Textbox 1 displays the inclusion and exclusion criteria that will be followed.

**Inclusion and exclusion criteria.**

**Inclusion criteria**

- Individuals with disabilities resulting from neurological, trauma, musculoskeletal or orthopaedical disorders, or visual or hearing impairments
- Be aged 18 years or older
- Daily use of 1 or more AT (eg, wheelchair, walking sticks, walking aid or walker, external prosthesis, hearing aid, visual aid, or orthosis) for at least 6 months
- Be able to complete questionnaires in French or participate in support interviews to complete them

**Exclusion criteria**

- Presence of preexisting psychological conditions before using ATs
- Use of ATs for a short period or irregularly

### Sample Size

The planned sample size for this study was calculated using the Cochran formula with a margin of error of 5% and an unknown population of AT users. A total of 384 participants will need to be recruited to allow for the generalization of our future results. A description of how this estimate was calculated is provided in Multimedia Appendix 1. Given that there are 12 departments in Benin, a maximum of 32 potential participants could be recruited from each department. However, the number of participants to be recruited in each region will be weighted according to the density of each region. For example, in the most populous departments (ie, urban and semiurban cities), more than 32 participants could be recruited, and in the least populous departments (ie, villages and small cities), fewer than 32 participants could be recruited on an equitable basis to achieve the desired sample size.

### Data Collection and Procedures

Once identified, AT users will be contacted by phone or met in person (at their homes or in rehabilitation centers) to receive a brief explanation of this study’s project. Participants who wish to take part in this study will receive a detailed information letter describing this study’s protocol. Assessments will be scheduled according to participants’ availability and will last approximately 1 hour. Regular breaks will be scheduled to minimize the risk of fatigue during assessments.

First, a form will be used to collect sociodemographic data from participants, including age, sex, gender, and level of education, as well as information about their use of ATs.The form is based on specific items from the rATA (rapid assistive technology assessment) developed by the WHO and published in 2022. The goal of the rATA is to provide a quick assessment of the needs, demands, supply, and satisfaction of AT users [30].

**Figure 1:**
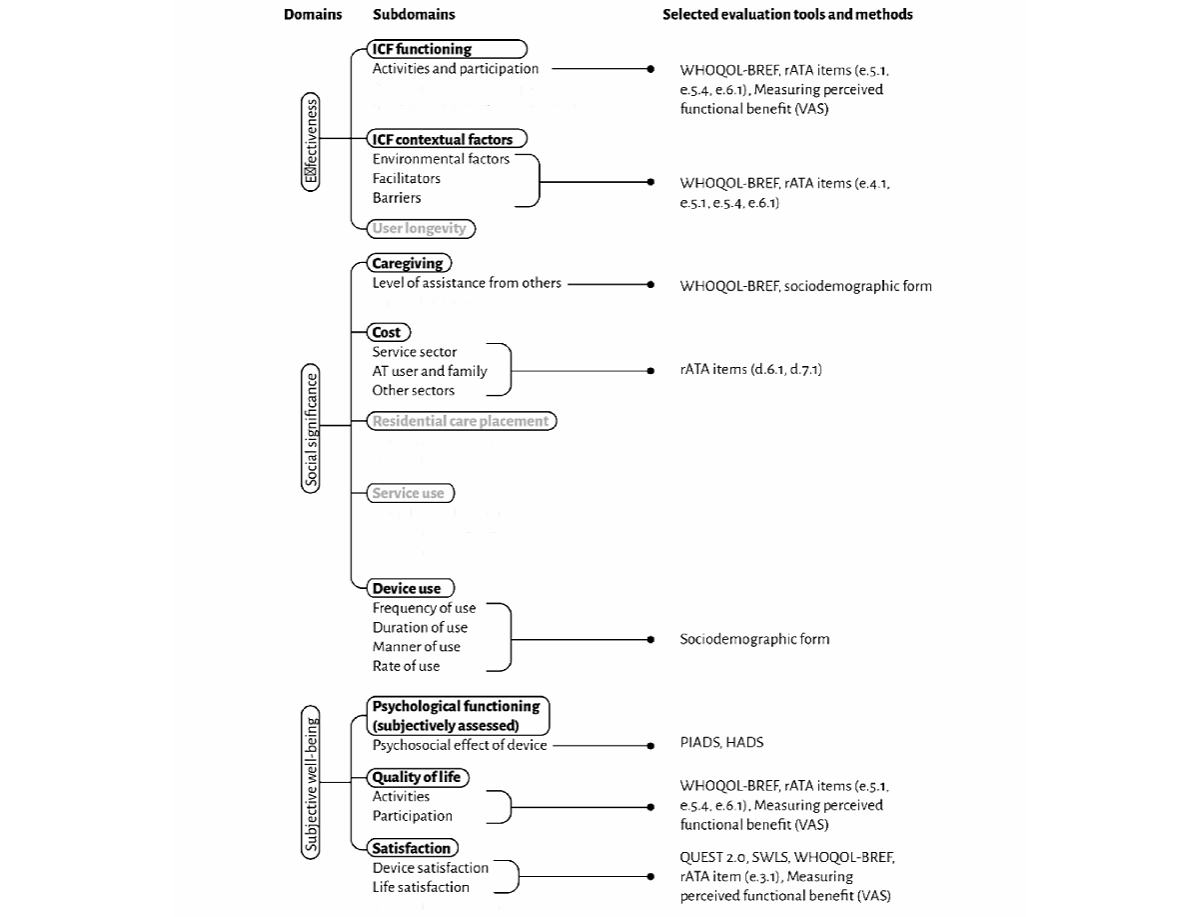
Selection of evaluation tools and methods based on the CATOR taxonomy. AT: assistive technology; CATOR: Consortium on Assistive Technology Outcomes Research; HADS: Hospital Anxiety and Depression Scale; ICF: International Classification of Functioning, Disability and Health; PIADS: Psychosocial Impact of Assistive Devices Scale; rATA: rapid assistive technology assessment; VAS: visual analog scale; WHOQOL-BREF: World Health Organization Quality of Life.

Second, the participants will undergo assessment using standardized tools or methods to determine their psycho-affective status, satisfaction with the assistive devices, perception of the functional effects of the devices, well-being, and QoL. These outcome measures have been selected from among the most widely used worldwide, according to the different domains and subdomains of the CATOR taxonomy (Figure 1 [8]). The following section describes the selected instruments:

- Psychosocial Impact of Assistive Devices Scale (PIADS): The French version of the PIADS will be used in this study [31]. This 26-item self-report questionnaire aims to evaluate the effects of ATs on functional independence, well-being, and QoL [32]. It was developed in response to the need for a simple, reliable, and valid scale that could be applied generically to all categories of AT [25]. The PIADS is an appropriate instrument for developing and testing theories regarding the psychosocial factors that may influence the use of AT [25]. It is widely used in clinical and research settings due to its ability to detect major changes in the acceptability of ATs and to predict their cessation or discontinuation [22-24]. A recent systematic review has demonstrated the robustness of the PIADS, with satisfactory conclusions drawn regarding its psychometric properties, including content validity, structural validity, internal consistency, criterion validity, test-retest reliability, and construct validity [18]. It is composed of three subscales: Competence: 12 items describing the user’s perceived functional capacity, independence, productivity, and performance [20,32]; Adaptability: 6 items that measure an individual’s motivation to take risks, seize opportunities, and try unusual things [20,32]; and Self-esteem: 8 items reflecting self-confidence, feelings of power and control, and emotional well-being [20,32]. Each item is evaluated using a 7-point Likert scale, ranging from –3 for the most negative impact to +3 for the most positive impact. A score of 0 indicates no impact or change [32].
- Quebec User Evaluation of Satisfaction With Assistive Technology 2.0 (QUEST-2.0): It aims to measure satisfaction with a wide range of assistive devices and asks users how satisfied they are with the specific characteristics of their assistive device [17,33]. It comprises 12 questions, 8 of which assess the device’s characteristics, including weight, size, length, width, fit, safety, durability, ease of use, comfort, and effectiveness [17,33]. The remaining 4 questions evaluate satisfaction with the service provided, including appliance maintenance and repair, professionalism, and follow-up [17,33]. Participant satisfaction with the AT used is evaluated on a 5-point scale, ranging from “not at all satisfied” to “very satisfied.” Additionally, participants are required to select the three most important characteristics of the AT used. A recent systematic review assessed all available versions of QUEST, including QUEST 2.0, and concluded that it has good reliability and validity [17].
- Measurement of perceived functional benefit: The functional benefit of AT use will be measured according to the method of van der Heide and de Witte [19]. This will be based on the following 2 questions, using the visual analog scale graduated from 0 to 100. For example, if the AT involved is a wheelchair, the questions will be formulated as follows: (Q1) “How would you rate your ability to get around?” and (Q2) “Imagine you don’t have a wheelchair; how would you rate your ability to move around?” The difference between the 2 scores obtained for these 2 questions (Q1 and Q2) represents the perceived functional benefit of using AT. A score of 0 indicates no perceived functional benefit. A score between 0 and 50 indicates a moderate perceived functional benefit, while a score of 50 or higher indicates a significant perceived functional benefit.
- Satisfaction With Life Scale (SWLS): The French version of SWLS [34] by Diener and colleagues [35] will be used. It consists of 5 statements measuring general satisfaction with one’s own life on a scale of 1 to 7 points [34,35]. SWLS has demonstrated favorable psychometric properties, including construct validity, internal consistency, and reliability [35]. It is suitable for use with individuals of varying ages [35].
- Hospital Anxiety and Depression Scale (HADS): Anxiety and depression symptoms will be assessed using HADS, which comprises 14 items scored from 0 to 3. Seven questions relate to anxiety and 7 to depression, giving two scores with a maximum score of 21 [36]. HADS has also demonstrated good psychometric properties across different language versions [37].
- World Health Organization Quality of Life (WHOQOL-BREF): WHOQOL-BREF is one of the most used tools to assess a person’s QoL. It consists of 26 questions and assesses 4 domains: physical health, mental health, social relationships, and environment [38]. The scores for the different domains should be considered separately, as this questionnaire does not provide an overall score [38]. WHOQOL-BREF is widely used in clinical and research settings due to its reliability and validity [38].

In the case where participants fail to respond to all questions on the various questionnaires, they will be contacted without delay to ascertain the reason for this and to update the data accordingly. Otherwise, their data will not be included in the analysis.

### Ethical Considerations

The intended study was approved by the Research Ethics Committee of the University of Parakou in Benin (257/2024/CLERB-UP/P/SP/R/SA) and will be conducted per the prescribed guidelines. There will be no financial compensation for this study, and each participant will be required to sign a consent form before being assessed. All data collected as part of this study will be treated as strictly confidential. Participants will be identified by a number to preserve their anonymity. Files will be securely kept and destroyed after 5 years from the end of data collection. Personal information will not be disclosed in public documents, and all necessary precautions will be taken to avoid any disclosure.

### Data Analysis

The data from completed questionnaires will be entered into Microsoft Excel 2021 (Microsoft Corp) to create a database, which will then be analyzed using IBM SPSS Statistics (version 26; IBM Corp) software. The results will be presented in tables and figures to highlight the sample’s particularities. Descriptive statistics such as frequency, percentage, mean and SD, or median (IQR) will be used to summarize sociodemographic data. Student *t* test or one-way ANOVA will be used to compare normally distributed numerical variables. Pearson or Spearman correlation coefficients will be used to determine any relationships between variables. Multivariate linear regressions will be used to determine any associative links between the measured variables.

The main results of the assessments using the different outcome measures described above will be organized using the CATOR taxonomy on aspects relating to the effectiveness and social significance of the use of ATs, as well as the subjective well-being of users [8], as shown in Figure 1. The evaluations will be limited to the fully dark-written CATOR domains and subdomains (Figure 1). In CATOR domain 1 (effectiveness), the different impacts and implications of ATs use in everyday activities and participation in social or community life, as well as environmental barriers and facilitators, will be reported. A quantitative assessment of the level of assistance received from others in the installation and use of ATs will be reported in CATOR domain 2 (social significance). This will also include an evaluation of the cost, frequency, rate, duration, and manner of use of ATs. Finally, the CATOR domain 3 (subjective well-being) will encompass all the results of the assessment of the psychosocial impact of ATs, satisfaction with ATs and with life, and overall QoL. The relationships between the results obtained in the 3 domains will be investigated to gain a more comprehensive understanding of the profile of AT users in Benin.

## Results

The process of identifying potential participants began in August 2024, and data collection is scheduled to start in January 2025 and continue for 12 months.

## Discussion

### Relevance and Strengths of this Study

Given the growing importance attributed to worldwide data regarding the use of ATs by individuals with disabilities in their daily lives [2], it is imperative to assess these devices across diverse settings or environments to ascertain their real effects on the lives of these individuals, especially in resource-limited countries. Understanding these effects could be useful for selecting ATs, involving potential users in the decision-making process, and enhancing the accessibility of ATs in line with global priorities defined by WHO and UNICEF [2]. We therefore intend to evaluate the effects of ATs in Benin, a sub-Saharan African country where there are no data on AT users. To achieve this aim, an exhaustive research project has been drawn up based on the CATOR taxonomy and the use of standardized tools, the most widely used and documented in the literature. In addition, sociodemographic data will be gathered using specific items from the rATA, a recently developed and WHO-certified instrument designed to assess AT users worldwide [30].

The CATOR taxonomy provides a diversified classification of the findings of all forms of AT and is adaptable to all types of populations with disabilities. It provides a more objective view of the various effects of using ATs and leads to the identification of any problems encountered by users. Its use in our study guarantees a holistic structuring of our future results, which could easily be discussed with existing data in the literature.

### Limitations

It is worth noting a few limitations in the design of this study. One of these limitations is that it was not possible to plan the assessment of the users who will be included in this study on all the aspects described in the CATOR taxonomy. These include aspects such as body functions, longevity, use of care services, and use of long-stay residences (Figure 1). A more specific design is required to evaluate these different aspects. In addition, the impact of ATs on caregivers’ workloads will not be evaluated in depth. This is a topic that could be the subject of a future study. Another limitation is that the convenience sampling method to be used may limit the generalizability of our results. However, the fact that we plan to recruit participants from all regions of the country may strengthen the relevance of our future results.

### Dissemination Plan

After completing this study, the results will be disseminated through various ways, including oral and poster presentations at national and international scientific conferences. Additionally, at least 2 scientific articles will be published in peer-reviewed journals.

### Conclusion

This research project aims to contribute to the advancement of knowledge on ATs in sub-Saharan Africa, with a particular focus on Benin, where there are currently no data available. The findings of this study will constitute the inaugural investigation into the use of ATs in Benin. They may serve as a basis for future research in this field.

## Appendix

Multimedia Appendix 1 Cochran formula and sample size calculation.

## Abbreviations

AT: assistive technology
CATOR: Consortium on Assistive Technology Outcomes Research
FAPHB: Fédération des Associations des Personnes Handicapées du Bénin
HADS: Hospital Anxiety and Depression Scale
PIADS: Psychosocial Impact of Assistive Devices Scale
QoL: quality of life
QUEST: Quebec User Evaluation of Satisfaction With Assistive Technology
rATA: rapid assistive technology assessment
SWLS: Satisfaction With Life Scale
UNICEF: United Nations Children’s Fund
WHO: World Health Organization
WHOQOL-BREF: World Health Organization Quality of Life
