# Supplementary material for: Evaluating the Impact of Assistive Technologies on Individuals With Disabilities in Benin: Protocol for a Cross-Sectional Study
Source: JMIR Res Protoc. 2024 Dec 9;13:e60869. doi: 10.2196/60869 (PMC11667128; doi:10.2196/60869)
Supplement: Multimedia Appendix 1 [file resprot_v13i1e60869_app1.pdf]

Cochran formula and sample size calculation

$$n = \frac{\frac{z\alpha}{2}^{2} \times \rho \times (1-\rho)}{i^{2}}$$

n: Sample size

ρ: Proportion of people using ATs (ρ is unknown and was estimated at 50% according to the standards established by *Cochran*)

i: Margin of error = 5%, confidence level (95%)

$\frac{z\alpha}{2}$: Critical value on the reduced centered normal distribution for a risk of error α.

For α=5%, $\frac{z\alpha}{2}$ =1.96. $n = 1.96^2 \times 0.5 \times 0.5 / 0.05^2 = 384.16$
